# Management of Neurofibromatosis of the Nipple-Areolar Complex

**DOI:** 10.1155/2021/6622416

**Published:** 2021-08-10

**Authors:** Fahad Aljindan, Lamiaa Aljehani, Bayan Alsharif, Hatan Mortada

**Affiliations:** ^1^Plastic Surgery Department, King Abdullah Medical City, Makkah, Saudi Arabia; ^2^General Surgery Department, Al-Noor Specialist Hospital, Makkah, Saudi Arabia; ^3^General Surgery Department, Security Forces Hospital, Makkah, Saudi Arabia; ^4^Division of Plastic Surgery, Department of Surgery, King Saud University Medical City, King Saud University, Riyadh, Saudi Arabia; ^5^Department of Plastic Surgery & Burn Unit, King Saud Medical City, Riyadh, Saudi Arabia

## Abstract

Neurofibromatosis type 1 is an autosomal dominant disease having an incidence of 1 in 3000 individuals. It primarily involves the peripheral nervous system and usually presents with many neurofibromas. On rare occasions, NF1 can affect the breast and manifests as nipple-areolar complex extranipple (pseudopolythelia) like neurofibromas which can be disfiguring and sometimes cause pain and therefore need to be addressed surgically. We present a case of a 31-year-old female, who had multiple pedunculated neurofibromas around the nipple on both breasts for 3 years. These lesions were associated with mild pain and were increasing in size. Surgical excision was done while preserving the nipples bilaterally. NF1 primarily involves the peripheral nervous system and usually presents with a large number of neurofibromas. Several case series of patients with NF1 have been reported, but there are only a few published reports on neurofibromas of the nipple-areolar complexes. These lesions can be painful and cause cosmetic deformity. In our case, these lesions were approached by circumferentially excising the redundant nipple-areolar skin containing the neurofibromas, while isolating the nipple on a central ductal and vascular pedicle. In conclusion, the redundant nipple-areolar skin containing the neurofibromas can simply be approached by circumferential excision while preserving the nipple. This technique is simple, easy to perform, while it allows duct preservation and preserves cosmesis.

## 1. Introduction

Neurofibromatosis is an inherited genetic disorder that affects the brain, spinal cord, nerves, and skin. Neurofibromatosis type 1 (NF1) is one of the most common autosomal dominant disorders as it affects approximately 1 in 4000 individuals [1]. It is mainly characterized by the presence of café au lait spots and neurofibromas.

Neurofibromas of the breasts are quite rare manifestations of patients with NF1. In such cases, they occur on the nipple-areolar complexes [2, 3], and their frequency increases with age. Reviewing the literature, several cases of patients with NF1 have been reported, but only a few reports have specifically examined neurofibromas of the nipple-areolar complexes [4, 5].

## 2. Case Presentation

### 2.1. History

A 31-year-old female patient, who is medically free, came with multiple pedunculated lesions around the nipple on both breasts which were 1^st^ noticed three years ago.

These lesions were increasing in size, associated with mild intermittent pain, no itching, no change in surrounding skin color, and no history of nipple discharge. The patient denied any history of previous trauma. Her family history was insignificant. No history of weight loss, loss of appetite, fatigue, or night sweats.

The patient stated that she underwent correction of scoliosis of cervical and thoracic spine 5 years prior to coming to our clinic. She is not known to have any food or drug allergies.

### 2.2. Physical Examination

**Figure 1:**
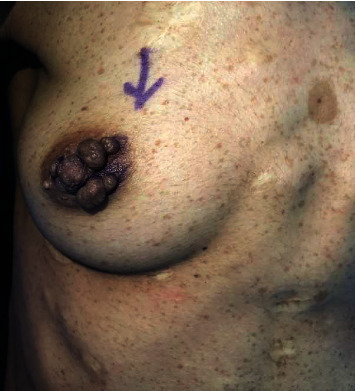
Multiple pedunculated lesions around right nipple preoperative.

**Figure 2:**
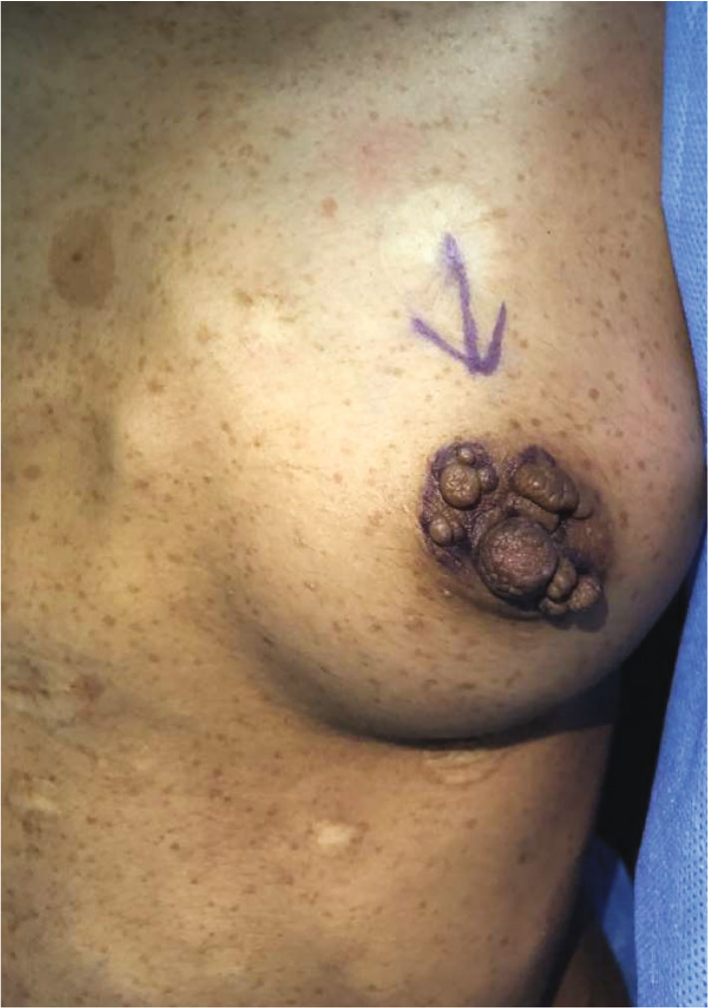
Multiple pedunculated lesions around left nipple preoperative.

On physical examination, the patient was conscious, alert, and oriented. Her vitals were stable but appeared to be underweight. She had multiple café-au-lait spots on her face, axillae, arms, and trunk. In addition, multiple pedunculated lesions were noticed around both nipples without noticeable changes in the surrounding skin, no palpable breast masses, no nipple discharge, and no palpable axillary lymph nodes (Figures 1 and 2). The left forearm showed a neurofibroma-like nontender lesion measuring about 0.5 × 0.5 cm and another similar lesion on her lower back lesion.

### 2.3. Laboratory Investigations

Preoperative blood workup was done and was within normal range.

The patient was seeking to have her nipple lesions excised. A decision was made to resect those lesions, and the patient consented to the procedure after a thorough discussion of all the possible pros and cons.

### 2.4. Procedure

**Figure 3:**
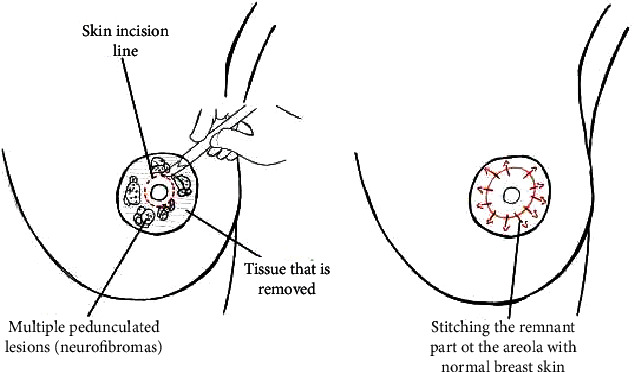
A drawing illustration explaining the marking of skin incision around the pedunculated lesions, followed by the circumferential and en bloc resection of the neurofibromas surrounding the nipples. Lastly, stitching the remnant part of the areola to normal skin.

With the patient lying supine under general anesthesia, both breasts were prepped and draped in the usual sterile manner with both arms abducted at 90 degrees. We started by isolating the nipple on each side on a central ductal pedicle using an 11 blade. This was followed by the circumferential and en bloc resection of the lesions surrounding the nipples. Finally, the intact areolar skin was approximated to the nipple on each side to recreate the nipple-areolar complex using 5.0 prolene (Figure 3). No complications were encountered, and blood loss was minimal. A layer of nonadherent dressing was applied, and the patient was discharged home on the same day in a stable condition.

### 2.5. Histopathology

Histopathology confirmed that the resected lesions were neurofibromas.

### 2.6. Follow-Up

**Figure 4:**
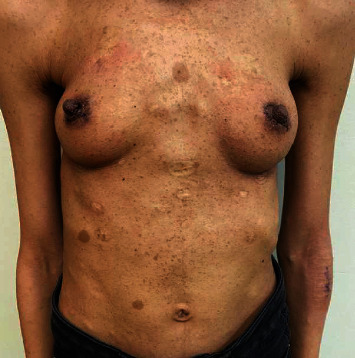
Postoperative day 7 showing healed bilateral nipple after lesion excision.

The patient was seen in the outpatient clinic a week later, and the nipple-areolar complex healed nicely without any vascular compromise. Sutures were removed on postoperative day 14 (Figure 4).

## 3. Discussion

NF1, also known as von Recklinghausen disease, is an autosomal dominant disease that primarily involves the neuroectodermal and mesodermal tissue. While the clinical manifestations of NF1 are well known, the course of the condition remains unpredictable.

NF1 primarily involves the peripheral nervous system and usually presents with a large number of neurofibromas.

On rare occasions, NF1 can affect the breast and manifests as nipple-areolar complex extranipple-like neurofibromas. Several case series of patients with NF1 have been reported, but there are only a few published reports on neurofibromas of the nipple-areolar complexes.

There are few cases in the literature describing invasive ductal carcinomas in association with von Recklinghausen disease. However, due to the paucity of reports of NF1, an association between these two types of diseases cannot be firmly established. Nevertheless, it is recommended that patients with neurofibromas of the breast should have a careful clinical and mammographic screening of the breast during adulthood to determine the presence or absence of malignancies [6]. Breast neurofibromas are uncommon NF1 manifestations that develop on the nipple-areolar complex [6]. According to the literature, women with NF1 have a higher risk of breast cancer than the general population; nevertheless, there are no special screening recommendations for these patients [7]. Thus, if cutaneous neurofibromas cause pain or are bothersome, they should be surgically removed [8], as in the presented case.

Our patient presented with severe neurofibromatosis of the nipple-areolar complex bilaterally without palpable breast masses. These lesions caused mild pain and deformity of her nipples and were approached by simple excision while preserving the duct. This was executed by circumferentially excising the redundant nipple-areolar skin containing the neurofibromas, while isolating the nipple on a central ductal and vascular pedicle. The nipple was finally sutured to the remaining areola using 5.0 prolene.

## 4. Conclusion

In patients with NF1 involving the nipple-areolar complex, the redundant nipple-areolar skin containing the neurofibromas can simply be approached by circumferential excision while preserving the nipple. This technique is simple, easy to perform, while it allows duct preservation and preserves cosmesis.
